# Interactions Between Heavy Trucks and Vulnerable Road Users—A Systematic Review to Inform the Interactive Capabilities of Highly Automated Trucks

**DOI:** 10.3389/frobt.2022.818019

**Published:** 2022-03-04

**Authors:** Victor Fabricius, Azra Habibovic, Daban Rizgary, Jonas Andersson, Pontus Wärnestål

**Affiliations:** ^1^ RISE Research Institutes of Sweden, Gothenburg, Sweden; ^2^ Halmstad University, Halmstad, Sweden; ^3^ Scania CV AB, Södertälje, Sweden

**Keywords:** truck, cyclist, pedestrian, interaction, automated driving system (ADS), heavy goods vehicle (HGV), vulnerable road user (VRU)

## Abstract

This study investigates interactive behaviors and communication cues of heavy goods vehicles (HGVs) and vulnerable road users (VRUs) such as pedestrians and cyclists as a means of informing the interactive capabilities of highly automated HGVs. Following a general framing of road traffic interaction, we conducted a systematic literature review of empirical HGV-VRU studies found through the databases Scopus, ScienceDirect and TRID. We extracted reports of interactive road user behaviors and communication cues from 19 eligible studies and categorized these into two groups: 1) the associated communication channel/mechanism (e.g., nonverbal behavior), and 2) the type of communication cue (implicit/explicit). We found the following interactive behaviors and communication cues: 1) vehicle-centric (e.g., HGV as a larger vehicle, adapting trajectory, position relative to the VRU, timing of acceleration to pass the VRU, displaying information *via* human-machine interface), 2) driver-centric (e.g., professional driver, present inside/outside the cabin, eye-gaze behavior), and 3) VRU-centric (e.g., racer cyclist, adapting trajectory, position relative to the HGV, proximity to other VRUs, eye-gaze behavior). These cues are predominantly based on road user trajectories and movements (i.e., kinesics/proxemics nonverbal behavior) forming implicit communication, which indicates that this is the primary mechanism for HGV-VRU interactions. However, there are also reports of more explicit cues such as cyclists waving to say thanks, the use of turning indicators, or new types of external human-machine interfaces (eHMI). Compared to corresponding scenarios with light vehicles, HGV-VRU interaction patterns are to a high extent formed by the HGV’s size, shape and weight. For example, this can cause VRUs to feel less safe, drivers to seek to avoid unnecessary decelerations and accelerations, or lead to strategic behaviors due to larger blind-spots. Based on these findings, it is likely that road user trajectories and kinematic behaviors will form the basis for communication also for highly automated HGV-VRU interaction. However, it might also be beneficial to use additional eHMI to compensate for the loss of more social driver-centric cues or to signal other types of information. While controlled experiments can be used to gather such initial insights, deeper understanding of highly automated HGV-VRU interactions will also require naturalistic studies.

## 1 Introduction and Background

How road space has been used, perceived, and designed has changed throughout history in response to new transportation technologies (e.g., trams, bicycles, and motorcars). Following each transition, a new set of rules and societal norms have emerged, which in turn has affected how road users are expected to behave within the traffic environment. The possible introduction of highly automated driving systems (ADS, i.e., level 3–5 in the Driving Automation taxonomy, Society of Automotive Engineers On-Road Automated Driving ORAD committee 2021), would arguably be one of the more impactful mobility innovations to influence the traffic environment. There are multiple scenarios in which these automated vehicles (AVs) could operate, including within: 1) Segregated AV networks, 2) Motorway or expressway networks, 3) Urban networks, or 4) Shared spaces (Parkin et al., 2018). While the first scenario could include occasional AV-human interactions (e.g., within a terminal- or construction area), it is the public contexts that highlight significant challenges in terms of AVs co-existing with humans. Consequently, there have been increasing research efforts to address how these novel road agents should behave around other road users.

However, while an automated driving system may be implemented for all types of vehicles, much of the research has focused on passenger cars (Dey et al., 2020). This paper extends the scope by including trucks, which due to their common use in a professional setting and the transportation of goods instead of passengers could be among the first AVs to reach widespread deployment (Illya Verpraet, 2021). More specifically, this study focuses on encounters and interactions between heavy goods vehicles (HGVs) (i.e., trucks in line with the European classification of a maximum permissible gross vehicle weight of over 3.5 tons) and vulnerable road users (VRUs). Current HGVs operate in diverse settings, and drivers are generally highly skilled at managing the rich set of situations they encounter in traffic. However, HGV related accidents still cause almost 4,000 fatalities every year in Europe (European Commission, 2016). Of these fatal accidents, 32% are reported to include VRUs and the majority involve pedestrians or cyclists (Kockum et al., 2017), which is also the reason for focusing this study on this particular group of VRUs.

While the safety perspective is generally one of the leading arguments for introducing AVs, these vehicles must also support appropriate traffic interactions in terms of traffic flow and road user experience. Since highly automated HGVs are not widespread in traffic today, there is a limited opportunity of studying their encounters and interactions with VRUs. Instead, this paper focuses on existing HGV-VRU studies, with the aim of understanding potential implications for future interactions between highly automated HGVs and VRUs. By conducting a systematic literature review, this paper addresses the following research questions:

• What interactive road user behaviors and communication cues can be identified in empirical HGV-VRU studies?
• What are potential implications for future interactions between highly automated HGVs and VRUs?

Before presenting the methodology, general findings, and synthesis from the review process, the following sections provide a background to the notion of road traffic interaction and (some of) its related theory and concepts.

### 1.1 Framing Road Traffic Interaction

It is important to highlight that many traffic situations involving two or more road users (i.e., traffic encounters) can unfold without resulting in interaction. Domeyer et al. (2020) use the more general term encounter to indicate when road users have a possibility of accommodating one another, with only one or neither adjusting their behavior. Similarly, they use the term interaction to indicate when both road users send signals that could be interpreted as their intent to accommodate one another or not. Markkula et al. (2020) trace existing theoretical perspectives of road traffic interaction to the following four categories: 1) traffic conflict and safety, 2) game theory, 3) sociology, and 4) communication and linguistics. Connected to these categories, researchers have studied road traffic interaction using different perspectives including collision avoidance, order of access, coordination, reciprocity, and communication. In an attempt to provide a more cross-theoretical framing of the notion, the authors first define the following two terms:

• **Space-sharing conflict:** An observable situation from which it can be reasonably inferred that two or more road users intend to occupy the same region of space at the same time in the near future.
• **Interactive behavior:** Road user behavior that can be interpreted as being influenced by a space-sharing conflict.

Based on this, they subsequently define road traffic interaction as follows:

• **Road traffic interaction:** A situation where the behavior of at least two road users can be interpreted as being influenced by a space-sharing conflict between the road users.

These are the definitions that are adopted for this study. However, it is acknowledged that the term interaction can be used in a variety of ways (Hornbæk and Oulasvirta, 2017; Hornbæk et al., 2019), suggesting that these definitions should be subject for future discussions.

#### 1.1.1 Interactive Scenarios in Road Traffic

**FIGURE 1:**
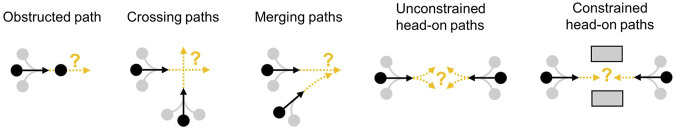
Prototypical space-sharing scenarios based on Markkula et al. (2020).

By focusing on space-sharing conflicts/scenarios as a basis for traffic interaction, interactive behaviors (and interactions) are more likely to occur in conjunction with two or more road users’ order of access to some shared region of space (Markkula et al., 2020). The authors argue that there is a limited number of ways two road users can approach a conflict space and that such situations can be generalized into five prototypical space-sharing scenarios, including obstructed paths, merging paths, crossing paths, and unconstrained and constrained head-on paths (Figure 1). Notably, when more than two road users are involved, multiple prototypes can apply simultaneously. While the simplicity of these prototypical scenarios support generalizations, other researchers have proposed more extensive taxonomies of scenes, situations, and scenarios that include more attributes and value facets (Ulbrich et al., 2015; Fuest et al., 2017).

#### 1.1.2 Road User Behavior and Communication

Observable behaviors in traffic are situated within a highly dynamic context. Domeyer et al. (2020b) adapted the transactional model of communication where road users are viewed as existing within “fields of experience” and relying on common ground (Clark and Brennan, 1991) as a basis for interaction and communication. They suggest that the degree of interdependence between road users will affect the need for interactive behaviors and communication. In earlier research (Johnson et al., 2014), interdependence has been defined as “the set of complementary relationships that two or more parties rely on to manage required (hard) or opportunistic (soft) dependencies in joint activity”. Here, the term joint activity is a generalization of joint action (Clark, 1996) and describes situations when what one party does depend on what another party does (and vice-versa) over a sustained sequence of actions. Johnson et al. (2014) suggest a “coactive design framework” (leveraging seminal work on teamworking principles) as an approach for supporting these hard and soft dependencies during joint activity. In brief, it has to do with supporting observability, predictability, and directability (OPD) between agents.

In their synthesis based on existing road traffic interaction literature, Markkula et al. (2020) highlight the tasks “moving” and “perceiving” as (the) two fundamental high-level tasks that road users perform to maneuver successfully in traffic. Furthermore, they state three basic types of behavioral effects in relation to these two tasks (“achieve”, “signal”, “request”), causing actions to have six different effects/impacts on the traffic situation. On top of this, they also identify a seventh (socially motivated) category of effects/impacts when road users signal appreciation. In terms of communication, road users’ various actions and behaviors can be classified into the following two main categories (International Organization for Standardization ISO, in progress):

• **Implicit communication:** Behavior that can be interpreted as serving the purpose of conveying information to another road user, but also as serving some other purpose (e.g., locomotion).
• **Explicit communication:** Behavior that can be interpreted as serving the exclusive purpose of conveying information to another road user.

Indeed, the mechanisms through which road users communicate are diverse and include both explicit cues, such as hand gestures and turning indicators, and more implicit cues, commonly conveyed *via* road users’ kinematic behaviors. These cues and signals can be further classified using theories of communication, such as the communicative aspect associated with nonverbal behavior (Domeyer et al., 2020b). Nonverbal behavior is a well-studied area with roots leading back to the 19th century (Darwin, 1873). While the exact definition may vary between research contexts, categories of nonverbal behavior include body movements and gestures, managing space and territory, touch, tone of voice, and appearance (Cowan et al., 1997). Table 1 summarizes these categories, where nonverbal behavior becomes nonverbal communication if another person interprets the behavior as a message and attributes meaning to it (Stefanov, 2018). Since this can be difficult to distinguish, we will use nonverbal behavior/communication interchangeably and include such sub-categories as part of a broader spectrum of possible channels/mechanisms that may also include modalities such as spoken language or text, signs, and symbols (e.g., various human-machine interfaces). While theories of nonverbal communication were originally developed for human (face-to-face) interactions, mechanisms such as vehicle turning signals have been likened to human facial expressions (Norman in Thomassen, 1994). Ultimately, universal questions of communication are concerned with accuracy, meaning, and effect of communication (Shannon, 1948).

**TABLE 1:** Categories of nonverbal behavior/communication as summarized by Stefanov (2018).

Categories of nonverbal behavior/communication	Description
Gestures and movement	This type of behavior is often called body language, and the study of the communicative aspects of all gestures, eye behaviors, facial expressions, posture, and movements of the hands, arms, body, head, legs, feet, and fingers is called kinesics
Space	The study of the communicative aspects of space and distance is called proxemics. Proxemic distances can be grouped into several categories including, public, social, personal, and intimate distance. The concept of territoriality groups spaces into several categories, including primary, secondary, and public spaces
Time	The study of the communicative aspects of time is called chronemics. Time can be grouped into several categories including, biological, personal, physical, and cultural time
Voice	Paralanguage refers to the vocalized but nonverbal part of the communication. The study of the communicative aspects of voice including, pitch, volume, rate, vocal quality, and verbal fillers, is called vocalics
Face and eyes	We also communicate through eye behaviors, primarily eye contact and face behaviors, primarily facial expressions. While face and eye behaviors are often studied under the category of kinesics, communicative aspects of eye behaviors have their own branch of studies called oculesics
Touch	The study of the communicative aspects of touch is called haptics. Touch is important for human social development, and it can be grouped into several categories including, welcoming, threatening, and persuasive touch
Appearance	Appearance involves physical characteristics and artifacts. There are many aspects of physical appearance that can potentially produce messages including, attractiveness, body size, body shape, facial features, hair, skin color, height, weight, clothing, watches, and necklaces
Environment	Environmental factors include architecture, interior spatial arrangements, music, color, lighting, temperature, scent, and smell. The study of the communicative aspects of scent and smell is called olfactics

The more recently sparked interest in interactions between AVs and other road users has influenced additional research to investigate existing traffic interaction practices (e.g., Dey and Terken, 2017; Lee et al., 2021), as well as the potential implications of introducing AVs into the public traffic environment (e.g., Lundgren et al., 2017; Rettenmaier and Bengler, 2021; Tabone et al., 2021).

#### 1.1.3 Factors Influencing Vehicle-VRU Interactions

Road user behavior is influenced by elements such as infrastructure, traffic rules, and cultural expectations (Renner and Johansson, 2006) and can include actions that are more strategic in nature (aligning with a game-theoretic perspective) or that arise in less calculated ways. Based on a meta-analysis of pedestrian negotiation and decision-making in roadway crossings, Rasouli and Tsotsos. (2019) synthesized a figure depicting a complex web of influential factors and sub-factors including pedestrian-centric factors (speed, attention, past experiences), and environmental factors (traffic flow, weather conditions, road infrastructure). Similarly, Madigan et al. (2019) concluded that the level and criticality of interactions between vehicles and VRUs is influenced by three broad factors—environmental/situational characteristics, road user characteristics, and vehicle characteristics. While the interrelationship within and between these factors will vary depending on situation and studied phenomena (e.g., crossing decision, gap acceptance, yielding behavior), they clearly range across the interactional, relational, and societal level illustrated in the adapted transactional model of communication proposed by Domeyer et al. (2020b).

#### 1.1.4 Summary and Implications for This Study

This brief theoretic background suggests that road traffic interactions may be viewed as short episodes of joint activity with the (subjectively assessed) presence of interactive behaviors aimed at resolving space-sharing conflicts between at least two road users. In these highly dynamic and context-dependent situations, actor may use implicit and explicit communication to seek various effects/impacts connected to the tasks of moving and perceiving in the traffic environment. Road users can also signal appreciation. Coordination devices such as rules, norms, and traffic control devices limit the degree of interdependence (and need for communication) among road users, while more ambiguous situations include negotiation and coordination. Interactive behaviors and communication cues can be linked to established theory of communication, where actions sometimes are intended and interpreted as signals and sometimes available as less deliberate cues for other road users to judge (or possibly misjudge). The following part of the paper leverages these theoretical concepts and definitions to guide the HGV-VRU literature review process and to structure the findings.

## 2 Methods

The literature search and selection process was based on the Preferred Reporting Items for Systematic Reviews and Meta-Analysis statement (Moher et al., 2015).

### 2.1 Literature Sources and Search Strategy

The search for studies was conducted through the databases Scopus, ScienceDirect and Transport Research International Documentation (TRID), using the search string (“truck” OR “HGV” OR “lorry”) AND (“behavior” OR “interaction” OR “communication” OR “conflict”) AND (“pedestrian” OR” cyclist” OR “vulnerable”) based on title, abstract, and keywords. The search was conducted in May 2021, with alerts for new publications continuing through to September 2021. Only documents published in English were considered, resulting in a total of 372 records (223 Scopus, 54 ScienceDirect, 95 TRID).

### 2.2 Study Selection Process

After removing duplicate entries, the records (*n* = 270) were screened for relevance by evaluating titles and abstracts, after which the exclusion process continued by evaluating full-text articles (*n* = 34) for eligibility. Both steps were guided by the following exclusion criteria:

• Studies with an unspecific type of heavy vehicle/driver or other than HGV (e.g., “drivers”, “heavy vehicles”, “vans”, “buses”, “forklift trucks”).
• Studies not focusing on road traffic encounters/interactions between road users (e.g., vehicle emissions, road infrastructure, driver health issues)
• Studies focusing on safety measures (e.g., blind-spot detection, front-end/sideguard design, VRU high-visibility clothing).
• Studies on accident frequencies and injury severity.
• Studies on methods, simulations, and modelling (e.g., simulator development, data collection techniques, traffic models/simulations).
• Studies with an unspecific type of VRU or other than pedestrians or cyclists (e.g., “motorcycles”, “mopeds”, “e-bikes”).
• Studies based on secondary data or stated preference (e.g., database analysis, meta-analysis, focus groups, surveys).

**FIGURE 2:**
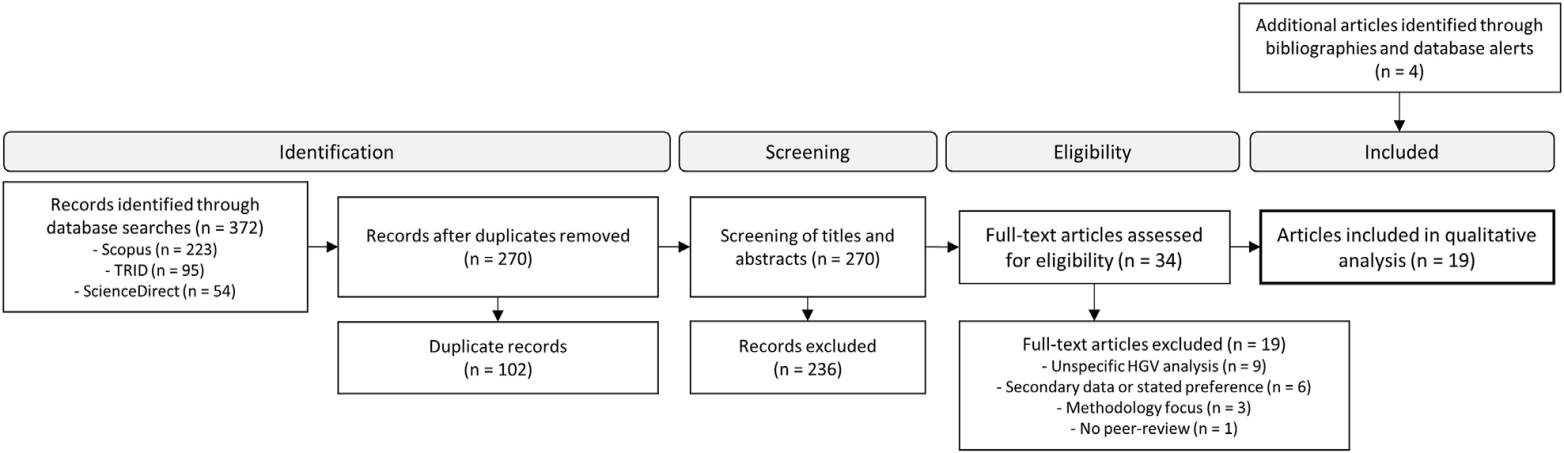
Search and selection flow diagram.

Apart from the more obvious criteria when searching for HGV-VRU interaction/behavioral studies (e.g., excluding other types of vehicles, studies on driver health issues, method development etc.), this list include additional delimitations. The choice was made to focus on pedestrians and cyclists, even if the term VRU sometimes refers to other groups such as motorcycles and powered two-wheelers (PTWs). In addition, we excluded studies based on secondary data or stated preferences. This was done due to the subjective nature of investigating interactive behaviors and communication (highlighted in the theoretical background) motivating first-hand sources with observations and analyses by researchers of the individual studies. The full-text eligibility step yielded 15 studies, and after additions from bibliographies and database alerts (*n* = 4), the selection included a total of 19 empirical HGV-VRU studies (see flow diagram in Figure 2).

### 2.3 Data Extraction and Analysis

After summarizing the basic characteristics of the included studies, they were categorized according to the type of investigated prototypical space-sharing scenario (presented in Section 1.1.1). From here, the analysis was of a qualitative nature summarizing study insights and extracting reports of interactive behaviors and communication cues of the included road users. Three of the paper authors (VF, DR, AH) independently reviewed the sample and later consolidated their findings, where the analysis relied on the theoretic background provided in Section 1.1, including the definition of the term interactive behavior as “road user behavior that can be interpreted as being influenced by a space-sharing conflict”. The first author also categorized the extracted interactive behaviors and communication cues according to the type of communication channel/mechanism (e.g., nonverbal communication sub-category) as well as the general type of communication (i.e., more implicit or explicit), adhering to the theoretical background under Section 1.1.

## 3 Results

This section presents the main findings from the systematic literature review and qualitative data extraction. A description of the sample is followed by three sections structured according to the type of interaction scenario (i.e., prototypical space-sharing conflict) addressed in the included study.

### 3.1 Description of the Sample

Table 2 lists the 19 empirical HGV-VRU interaction/behavioral studies that met the eligibility criteria for this review. The studies originate from Europe (*n* = 14), the United States (*n* = 2), Australia (*n* = 2), and Asia (*n* = 1), and the majority are published during the last decade. The studies address HGVs in scenarios with cyclists (*n* = 15), pedestrians (*n* = 2), or both cyclists and pedestrians (*n* = 2). The methodologies and data collection approaches include controlled experiments (*n* = 9) (e.g., test track/simulator/virtual reality VR experiments), semi-controlled experiments (*n* = 6) (e.g., instrumented vehicle/bicycle field experiments), and naturalistic studies (*n* = 4) (e.g., naturalistic driving/riding studies, observational studies).

**TABLE 2:** Basic characteristics of the included studies.

Author(s), year, location	Title	Objective	Method/data collection	Sample size	Interactants
Abadi et al. (2019), US	Factors impacting bicyclist lateral position and velocity in proximity to commercial vehicle loading zones: Application of a bicycling simulator	Do engineering treatments (markings and signs) and truck maneuver have any effect on the bicyclists’ velocity and lateral position in the bicycling environment?	Bicycle simulator experiment	48 participants	HGV-cyclist
Beck et al. (2019), AU	How much space do drivers provide when passing cyclists? Understanding the impact of motor vehicle and infrastructure characteristics on passing distance	Quantify passing distance and assess the impact of motor vehicle and road infrastructure characteristics	Naturalistic riding study	60 participants, 379 overtakes by trucks	HGV-cyclist
Beck et al. (2021), AU	Subjective experiences of bicyclists being passed by motor vehicles: The relationship to motor vehicle passing distance	Explore the relationship between cyclists’ subjective experiences and the lateral passing distance of motor vehicles	Naturalistic riding study	60 participants, 379 overtakes by trucks	HGV-cyclist
Chuang et al. (2013), TW	The use of a quasi-naturalistic riding method to investigate bicyclists’ behaviors when motorists pass	Investigate how motorized vehicle-related factors, road-related factors, and bicyclist-related factors influence passing events	Instrumented bicycle experiment	34 participants	HGV-cyclist
Colley et al. (2020), DE	Evaluating Highly Automated Trucks as Signaling Lights	Investigate interactions and external communication when an automated truck is blocking a sidewalk	Virtual Reality experiment	20 participants	Highly automated HGV-pedestrian
Dozza et al. (2016), SE	How do drivers overtake cyclists?	Explore overtaking scenarios and quantify the corresponding driver comfort zones	Instrumented bicycle experiment	10 overtakes by trucks	HGV-cyclist
Garcia et al. (2020), ES	Influence of peloton configuration on the interaction between sport cyclists and motor vehicles on two-lane rural roads	Investigate risks associated to the interaction with motor vehicles of cyclists riding in a peloton	Instrumented bicycle experiment	73 overtakes by trucks	HGV-cyclist
Jashami et al. (2020), US	The Impact of Commercial Parking Utilization on Cyclist Behavior in Urban Environments	Evaluate the impact of commercial vehicle loading and unloading activities on safe and efficient bicycle operations in a shared urban roadway environment	Bicycle simulator experiment	48 participants	HGV-cyclist
Kircher and Ahlström (2020), SE	Truck drivers’ interaction with cyclists in right-turn situations	Investigate truck drivers’ speed choice, gaze behaviour, and interaction strategies in relation to VRUs when turning right in signalized and non-signalised intersections	Semi-controlled naturalistic experiment	29 participants	HGV-Cyclist
Kircher et al. (2020), SE	Effects of training on truck drivers’ interaction with cyclists in a right turn	Explore the effects of training truck drivers in anticipatory driving to improve their interaction with cyclists	Semi-controlled naturalistic experiment	15 participants	HGV-Cyclist
Petzoldt. (2016), DE	Size speed bias or size arrival effect—How judgments of vehicles’ approach speed and time to arrival are influenced by the vehicles’ size	Clarify the relationship between size speed bias and size arrival effect	Video experiment	39 participants	HGV-VRU
Petzoldt et al. (2017), DE	Time to Arrival Estimates, (Pedestrian) Gap Acceptance and the Size Arrival Effect	Investigate whether the size arrival effect that is prevalent in time to arrival estimates can explain the variations in gap acceptance	Video experiment	27 participants	HGV-pedestrian
Pitera et al. (2017), NO	The complexity of planning for goods delivery in a shared urban space: a case study involving cyclists and trucks	Examine issues related to freight delivery on a street section with a high volume of cyclists	Video observational study	1,358 observations	HGV-cyclist
Pokorny and Pitera (2019), NO	Observations of truck-bicycle encounters: A case study of conflicts and behaviour in Trondheim, Norway	Exploring the behaviors and conflicts surrounding truck–bicycle encounters	Video observational study	979 encounters, 31 conflicts	HGV-cyclist
Richter and Sachs (2017), DE	Turning accidents between cars and trucks and cyclists driving straight ahead	Investigate driving and gaze behavior during right turning	Truck simulator experiment	48 participants	HGV-cyclist
Schindler and Bianchi Piccinini (2021), SE	Truck drivers’ behavior in encounters with vulnerable road users at intersections: Results from a test-track experiment	Assess how HGV drivers negotiate the encounters with VRUs in two scenarios	Test-track experiment	13 participants	HGV-VRU
Thorslund and Lindström (2020), SE	Cyclist strategies and behaviour at intersections. Conscious and un-conscious strategies regarding positioning	Examine the typical behavior among cyclists in terms of positioning themselves when passing an intersection	Bicycle simulator experiment	33 participants	HGV-cyclist
Twisk et al. (2018), NL	Higher-order cycling skills among 11- to 13-year-old cyclists and relationships with cycling experience, risky behavior, crashes and self-assessed skill	Assess the level of higher-order cycling skill among children	Video experiment	335 participants	HGV-cyclist
Walker (2007), United Kingdom	Drivers overtaking bicyclists: Objective data on the effects of riding position, helmet use, vehicle type and apparent gender	Present behavioral data on drivers’ overtaking around bicyclists	Instrumented bicycle experiment	A total of 2,355 vehicle overtakes	HGV-cyclist

### 3.2 Interactive Behaviors in Obstructed Path Scenarios

From the 19 identified empirical HGV-VRU studies, 10 could be linked to obstructed path scenarios such as the road being blocked by trucks or overtaking situations. Several researchers have analyzed HGV encounters as part of wider data collection on vehicle-cyclist passing events. Using an instrumented bicycle, Walker (2007) found that large vehicles pass cyclists significantly closer as compared to smaller vehicles (reporting a mean overtaking proximity of 1.15 m). Owing to their length and poor acceleration, large trucks take much longer to pass a cyclist than shorter vehicles. To pass safely, a driver must encroach onto the oncoming traffic lane for an extended period (even with a cyclist riding towards the road edge). It is suggested that cyclists should acknowledge the overtaking limitations of long vehicles in urban environments and assist their overtaking efforts where practicable. Chuang et al. (2013) found that longer passing times caused the cyclists to exhibit more cautious and less stable cycling behavior while the motorists passed. In another study using instrumented bicycles, Dozza et al. (2016) investigated how drivers overtake cyclists on rural roads. During this maneuver, drivers regulate speed and lateral position, negotiating with potential oncoming traffic to stay within their comfort zones while approaching and passing cyclists. They identified four overtaking phases (approaching, steering away, passing, and returning) and quantified the corresponding driver comfort zones. Three overtaking strategies were considered: 1) the flying strategy, where drivers overtake cyclists while keeping their speed relatively constant, 2) the accelerative strategy where drivers slow down and follow the cyclists for some time before passing, and 3) the piggybacking strategy adopted by drivers who follow a lead vehicle. While the sample size of HGVs was small, comfort zone boundaries were found to be longer for trucks than cars only in the approaching phase, and the trucks spent more time in the passing phase. Garcia et al. (2020) found that passing vehicle speeds were lower when cyclists (racers) were riding in a group, and that HGVs had lower lateral clearance. Cyclists’ subjective risk perception was negatively affected by increased vehicle speed, decreased clearance, and larger vehicle size (referencing the aerodynamic forces that an overtaking vehicle produces). Beck et al. (2019) instrumented participants’ own bicycles in their naturalistic riding study. Overall, one in every 17 passing events was a close (<100 cm) passing event, and they identified that road infrastructure (specifically on-road cycle lanes) had a substantial influence on the distance that motor vehicles provide when passing cyclists. Based on the same dataset, Beck et al. (2021) also investigated the subjective experiences of cyclists being passed by motor vehicles. Using a “panic button” on the instrumented bicycles, they found that the proportion of passing events with a recorded button press were over three-fold higher in events where the cyclist was passed by an HGV (3.7%) compared to a sedan (0.9%). Across all conditions, the predicted probability of a button press was 1% at a passing distance of 140 cm, 6% at 100 cm and 23% at 60 cm, and the study concluded an increased perceived risk in events where cyclists were passed by large vehicles such as HGVs.

In an observational study, Pitera et al. (2017) found that cyclists tended to adapt their behavior and trajectory depending on the position of a parked HGV in relation to the cycle lane. More specifically, when passing an HGV parked in a loading zone, the cyclists adopted one of the following behaviors: 1) continue using the cycle lane, 2) riding around using the sidewalk, or 3) riding around using the road. The two latter behaviors occurred when the truck was blocking the cycle lane; half of the cyclists adopted behavior b) and half of them adopted behavior c). Similar behavioral adaptations were observed in situations when the HGV was reversing, which made nearly half of the cyclists react in some way (e.g., riding in the opposite traffic lane, going around the reversing truck, waiting in the cycle lane while the truck was reversing). Related insights are provided by Jashami et al. (2020), who concluded that larger loading zones for trucks in the proximity of cyclists resulted in the cyclists adopting slower speed and greater lateral distances from the loading zone even when the zone size was not directly obstructing the trajectory of the cyclists. Pokorny and Pitera (2019) reported that in a scenario where HGVs and cyclists will continue after having stopped at the red phase at traffic lights, cyclists accelerated faster than and thus “escaping” the trucks’ proximity. The study further noted that cyclists’ waiting positions in these static scenarios varied, and that cyclists in the presence of HGVs tended to select the most visible positions.

Colley et al. (2020) conducted a virtual reality VR experiment with a scenario where a highly automated HGV was blocking a sidewalk. They tested different types of explicit communication (*via* external human-machine interfaces, eHMI) to provide supporting information for approaching pedestrians, using symbols, text, colors, auditory signals, and other displayed features on the HGV. Based on their experiment they concluded that the information of being able to walk safely past the truck was highly appreciated by the test participants.

To summarize, from the reviewed studies on obstructed path scenarios, we identified several examples of HGV-VRU interactive behaviors and communication cues (Table 3). A great majority of these were classified as implicit communication as defined in Section 1.2.1. More specifically, two of these implicit cues are related to the appearance and characteristics of HGVs (e.g., large/heavy, often driven by a professional driver) and VRUs (e.g., unprotected, wearing helmet, gender) that may set expectations and affect behavior in terms of clearance and acceleration (Walker, 2007). The rest of the implicit cues reflect communication *via* movement, position, and timing (kinesics, proxemics, and chronemics nonverbal behavior): HGV driver adopting a “flying”, “accelerative”, or “piggybacking” strategy when overtaking a cyclist (Dozza et al., 2016), HGV passing a VRU in close proximity (Chuang et al., 2013; Beck et al., 2019; Garcia et al., 2020; Beck et al., 2021), cyclists adapting their trajectory depending on the position of the HGV (Pitera et al., 2017; Jashami et al., 2020), pedestrian passing the obstructing HGV by stepping onto the roadway (Colley et al., 2020). When it comes to explicit communication, one of the cues identified is based on strategic positioning (proxemics) to request perception and involves cyclists selecting a more visible position when the HGV is present (Pokorny and Pitera, 2019), while the other one involves HGVs displaying colors, symbols, and text to VRUs in their vicinity *via* eHMI (Colley et al., 2020). Notably, several of the HGV-VRU interaction patterns might differ from corresponding interactions between VRUs and light vehicles: as compared to light vehicles, HGVs (and other large vehicles associated with professional drivers) displayed closer proximity when overtaking cyclists (Walker, 2007; Beck et al., 2019), took longer to overtake (Walker, 2007; Dozza et al., 2016), and made cyclists feel less safe (Garcia et al., 2020; Beck et al., 2021).

**TABLE 3:** Reported interactive road user behaviors/communication cues from HGV-VRU obstructed path scenarios, including their motivation/effect, communication channel/mechanism, type of cue, and reference.

Road user behavior/communication cue	Motivation/effect	Communication channel/mechanism	Type of communication cue	References
HGV characteristics (large/heavy vehicle often driven by a professional driver). VRU characteristics (e.g., unprotected, wearing helmet, gender)	Sets expectations and may affect interaction capabilities and patterns	Appearance	More implicit cue	Walker (2007)
Adopting a “flying”, “accelerative”, or “piggybacking” strategy when overtaking the cyclist	The driver seeking to stay within their comfort zone	Kinesics, proxemics, chronemics	More implicit cue	Dozza et al. (2016)
HGV passing VRU in close proximity	Passing distance below 1 m considered a close passing event	Kinesics, proxemics	More implicit cue	Chuang et al. (2013), Garcia et al. (2020), Beck et al. (2019), Beck et al. (2021)
Cyclists adapting their trajectory depending on the position of the blocking truck in relation to the infrastructure (loading zone, cycle lane, sidewalk)	Anticipating people or objects emerging	Kinesics, proxemics, environment	More implicit cue	Pitera et al. (2017), Jashami et al. (2020)
Pedestrian passing the obstructing truck by stepping onto the roadway	Movement-achieving	Kinesics, proxemics, chronemics	More implicit cue	Colley et al. (2020)
Truck external human-machine interface (eHMI) displaying colors, symbols, and text	Provide information to VRUs	Human-machine interface	More explicit cue	Colley et al. (2020)
Cyclist selecting a more visible position when HGV is present	Avoid blind-spot (i.e., perception-requesting behavior)	Proxemics	More explicit cue	Pokorny and Pitera (2019)

### 3.3 Interactive Behaviors in Crossing Paths Scenarios

Nine of the empirical HGV-VRU studies could be linked to crossing paths scenarios such as road crossings. In an observational study from signalized intersections in Norway, Pokorny and Pitera (2019) showed that most HGV drivers (78%) selected “safer” positions further back from the stop line (distance >1 m) when a cyclist was present. This behavior was explained by the HGV drivers’ aspiration for gaining a better overall view of the area while considering potential blind-spots. Results also showed that HGV drivers are used to stopping for cyclists even if the drivers hold the right of way. This was even more common for passenger cars, possibly explained by the fact that decelerating and accelerating is more demanding for HGVs. In addition, cyclists would more often dismount their bicycle (leading to priority at a pedestrian crossing) where the road was wider and the speed of the HGV was higher, and any negotiations would typically end with the cyclist waving their arm to thank the truck driver. In a semi-controlled study where HGV drivers used eye-tracking equipment and an instrumented vehicle, Kircher and Ahlström (2020) reported that glances towards cyclists in right turn intersection scenarios were more frequent when the intersection included greater distances than shorter distances. In situations where there was free-flowing traffic, the HGV drivers glanced less towards the cyclist, possibly due to having better chances to choose safer interaction strategies such as staying behind the cyclist. The authors describe typical ways of how an HGV-VRU turning/crossing scenario might unfold depending on various situational aspects (e.g., road user trajectories, infrastructure layout, traffic control devices, presence of other traffic), and where the interaction ends with “either the truck or cyclist going first”. In another study with a similar methodology (Kircher et al., 2020), improved driver behavior from before and after training could be observed, such as better speed management, strategic/tactical positioning strategies, and more intensive monitoring of cyclists. The authors state that adopting such anticipatory driving techniques can improve interactions with VRUs. Richter and Sachs (2017) also studied right-turning HGVs and cyclists going straight, finding that HGV driver’s relative gaze frequency to the cyclist through the right window increased when the distance between the lanes decreased.

Thorslund and Lindström (2020) used a cycle simulator to investigate cyclists’ conscious and unconscious strategies regarding positioning at intersections in mixed traffic. With the HGV present, participants rode slower, kept more to the left, as well as stopped farther from the stop line. The most frequent strategic considerations were to obtain a good overview, visibility, avoid blind-spots, and be prepared for the vehicle turning right without the use of turning indicators. As part of a longer video-based experiment, Twisk et al. (2018) investigated 11- to 13-year-old cyclists’ preferred behaviors during encounters with an HGV waiting at a signalized intersection. They found that the participants often selected dangerous positions (i.e., blind-spots) relative to the HGV. Furthermore, the authors argue that limitations in higher-order skills may be detrimental for the safety of youngsters, and these children appear to overestimate their level of skill, which may contribute to over confidence, violations, and errors. In a test track experiment, Schindler and Bianchi Piccinini (2021) found that truck drivers adapted their kinematic and visual behavior in a crossing when pedestrians and cyclists were present. Compared to the baseline (no VRU), the speed profiles of the drivers diverged approximately 30 m from the intersection and glances were directed more often towards front right and right when the cyclist was present. For the scenario with a pedestrian crossing, the drivers changed their speed about 14 m from the intersection and glances were directed more often towards the front center.

The aim of Petzoldt’s (2016) experiment was to clarify the relationship between the contradicting size speed bias (i.e., the phenomenon that observers underestimate the speed of larger objects) and size arrival effect (i.e., that larger objects are judged as arriving earlier than smaller ones). The results confirmed the size speed bias for the speed judgments, with the HGV being perceived as travelling slower than a car. Referencing several sources that have found motorists to be consistently more conservative when confronted with larger vehicles, it was suggested that factors other than perceived speed or time-to-collision TTA play an important role for the differences in gap acceptance between different types of vehicles such as expected cost/consequence of an accident. In a following controlled video experiment, Petzoldt et al. (2017) found that vehicle size and perceived threat correlated substantially. However, it was unclear to what degree these factors contributed to pedestrian’s crossing decisions or perceived TTA.

To summarize, from the reviewed empirical studies on crossing path scenarios, we identified several examples of HGV-VRU interactive behaviors and communication cues (Table 4). Like the obstructed path scenarios (Section 3.2), a great majority of these fall into the category implicit communication. More specifically, two of them are related to the appearance and characteristics of HGVs that either contribute to poor situation awareness of cyclists in terms of choosing to stop in blind-spots of the HGV driver (Twisk et al., 2018), or affect expectations and behavior of cyclists in terms of gap acceptance (Petzoldt, 2016). Furthermore, one of the implicit cues reflects communication *via* relative position (chronemics): an HGV driver choosing to stop further from the stop line when a cyclist is present in order to ensure a sufficient safety margin to the cyclist (Pokorny and Pitera, 2019; Kircher and Ahlström, 2020). Three other implicit cues that we identified reflect communication *via* movements and proximity (kinesics and proxemics): pedestrians accepting a gap and deciding to cross the street (Petzoldt et al., 2017), an HGV driver considerably reducing the speed when encountering a VRU to signal his/her willingness to give way (Schindler and Bianchi Piccinini, 2021), and cyclists dismounting their cycles to get priority at a zebra crossing (Pokorny and Pitera, 2019). The rest of the implicit cues reflect a combination of eye/body language (oculesics/kinesics) (an HGV driver or cyclist directs his/her head and glances towards the interacting partner to get perception of the situation and possibly to signal a request for movement or perception, Kircher and Ahlström (2020), Kircher et al. (2020), Richter and Sachs (2017), and Schindler and Bianchi Piccinini (2021)), and a combination of kinesics, proxemics and chronemics [an HGV approaching a cyclist and choosing to remain behind in order to leave the opportunity for the cyclists to cross first, Kircher and Ahlström (2020)]. When it comes to the more explicit communication cues that we identified, one of them reflects selecting a strategic position (proxemics) to request perception: a cyclist stops earlier and more to the left in the lane to avoid blind-spots around the HGV and thereby enable the HGV driver to perceive him/her (Thorslund and Lindström, 2020). Cyclists will also wave their arm to say thanks (Pokorny and Pitera, 2019), and drivers use turning indicators to signal to cyclists in the vicinity (Thorslund and Lindström, 2020). It is also worth noticing that there seems to be a discrepancy in HGV-VRU interaction patterns in crossing path scenarios as compared to light vehicles. In particular, HGVs appear more threatening (Petzoldt et al., 2017), may be perceived as travelling slower (Petzoldt, 2016), and drivers will to a larger extent try to avoid decelerations and accelerations if they can (Pokorny and Pitera, 2019). Lastly, the biggest difference compared to most other vehicles is the more limited field of view causing larger blind-spots for the HGV driver.

**TABLE 4:** Reported interactive road user behaviors/communication cues from HGV-VRU crossing paths scenarios, including their motivation/effect, communication channel/mechanisms, type of cue, and references.

Road user behavior/communication cue	Motivation/effect	Communication channel/mechanism	Type of communication cue	References
HGV characteristics (large/heavy vehicle)	Sets expectations and may affect road users’ behavior such as gap acceptance	Appearance	More implicit cue	Petzoldt, (2016)
Cyclist approaching an HGV at an intersection	Cyclist aware/unaware of HGV blind-spots	Appearance	More implicit cue	Twisk et al. (2018)
Driver stopping farther from the stop line when a cyclist is present	Driver seeking overview and greater safety margin to VRUs	Proxemics	More implicit cue	Pokorny and Pitera (2019), Kircher and Ahlström (2020)
Cyclist dismounting bicycle at zebra crossing	Get priority as a pedestrian	Kinesics/proxemics	More implicit cue	Pokorny and Pitera, (2019)
Driver/cyclist glances towards other road users	Monitor the environment (i.e., perception-achieving behavior). Possible signal/request for movement or perception	Oculesics, kinesics	More implicit cue	(Kircher and Ahlström (2020), Kircher et al. (2020), Richter and Sachs (2017), Schindler and Bianchi Piccinini (2021)
Driver approaching cyclist and remaining behind	Leaving an opportunity for a cyclist to cross first	Kinesics, proxemics, chronemics	More implicit cue	Kircher and Ahlström (2020)
Pedestrian deciding to cross the street (accepting a gap)	Movement achieving, Possible signal/request for perception	Kinesics	More implicit cue	Petzoldt et al. (2017)
Driver considerably reducing speed when encountering a VRU	Movement achieving/signaling/requesting	Kinesics	More implicit cue	Schindler and Bianchi Piccinini (2021)
Cyclist waving arm	Thank driver after negotiation	Kinesics	More explicit cue	Pokorny and Pitera (2019)
Cyclist stopped earlier and more to the left in the lane	Avoid blind-spot (i.e., perception-requesting behavior)	Kinesics, proxemics	More explicit cue	Thorslund and Lindström (2020)
Driver using turning indicators	Movement signaling	Human-machine interface	More explicit cue	Thorslund and Lindström (2020)

### 3.4 Interactive Behaviors in Merging Scenarios

In a bicycle simulator experiment, Abadi et al. (2019) investigated interactions at loading zones incorporating different HGV behaviors (i.e., no truck, parked truck, truck pulling out) and infrastructure designs (varying road markings and warning signs). The results showed that truck presence does influence cyclist’s performance (i.e., velocity and lateral position), and this effect varies based on the design treatments employed. When a truck was present, cyclists had a lower velocity and lower divergence from the edge of the bike lane on solid green pavement, and a higher divergence from the edge of the bike lane when a warning sign was present.

From the study by Abadi et al. (2019), which was the only one containing a merging scenario, we identified two examples of HGV-VRU interactive behaviors and communication cues (Table 5). One of them is linked to implicit communication and reflects communication *via* movements and position (kinesics and proxemics): the presence of an HGV in a loading zone next to a cyclist lane makes the cyclists slow down and choose a lateral placement in the lane further away from the HGV. The other one is linked to explicit communication *via* traffic environment elements/signs: an HGV in a loading zone that is painted in green makes the cyclists slow down more and diverge less from the edge, while the presence of a warning sign makes them to diverge more from the edge. Altogether, this exemplifies that cyclists might adjust their behavior not only to the presence and anticipated behavior of the HGV, but also to cues in the traffic environment.

**TABLE 5:** Reported interactive road user behaviors/communication cues from HGV-VRU merging scenarios, including their motivation/effect, communication channel/mechanism, type of cue, and reference.

Road user behavior/communication cue	Motivation/effect	Communication channel/mechanism	Type of communication cue	References
Cyclist slowing down and moving to the side in the lane	HGV maneuver had a decreasing effect on velocity and an increasing effect on lateral position	Kinesics, proxemics	More implicit cue	Abadi et al. (2019)
Loading zone painted in patterns/colors and outfitted with signs	Indicate specific infrastructure element and potential hazard	Environment	More explicit cue	Abadi et al. (2019)

## 4 Discussion

This section contains a discussion based on the two research questions: 1) What interactive road user behaviors and communication cues can be identified in empirical HGV-VRU studies? 2) What are potential implications for future interactions between highly automated HGVs and VRUs?

### 4.1 Current HGV-VRU Interactive Behaviors

While we can observe behaviors and collect data using controlled, semi-controlled, and naturalistic studies, it is harder to interpret their influence or underlying motivation. Apart from the more general influencing factors described in Section 1.1.3, researchers have investigated factors based on HGV-VRU encounters. Influencing (safety) factors derived from HGV-cyclist literature include a lack of awareness regarding blind-spots, adopting risk taking behaviors (e.g., using phone while crossing/driving), and the lack of visual contact and communication between road users (Pokorny and Pitera, 2019). Examples of influencing factors derived from the reviewed studies on HGV-pedestrian interactions include blind-spot issues, size of traffic gap, and road users’ individual characteristics such as vehicle size or observed pedestrian age (Petzoldt et al., 2017; Naser et al., 2017). In addition, there will also be contextual influences including interaction at unfamiliar locations, objects limiting visibility, unsafe infrastructure layouts, and adverse weather conditions (Pokorny and Pitera, 2019; Sheykhfard and Haghighi, 2020). From the reviewed studies, we found several examples of how HGV-VRU characteristics are affecting encounters and interactions (Tables 3–5). However, there are also conclusions that the combination of infrastructure design and surrounding traffic was reported to have a larger impact on the development of the interaction between HGV and cyclist than the truck driver had (Kircher and Ahlström, 2020). The interconnected relationships between these factors are what contributes to the complex (or wicked) reality of the traffic domain.

So, while acknowledging this complexity, we do conclude that HGV-VRU interactive behaviors are shaped by the general characteristics of these road users. HGVs are among the larger and heavier vehicles on public roads, affecting vehicle dynamics and increasing the risk of severe outcomes in the event of an accident. They most often have professional drivers who need to handle large visual blind-spots and unpredictable, possibly inattentive, VRUs. The safety imbalance between these road users is indicated by reported behaviors including cyclists being more cautious and selecting strategic positions in the presence of an HGV (Pokorny and Pitera, 2019; Thorslund and Lindström, 2020), and drivers selecting a position with better overview or giving up right of way (Pokorny and Pitera, 2019). The reviewed studies more frequently report on road users’ kinematic behaviors, suggesting implicit communication to be the primary mechanisms facilitating HGV-VRU encounters and interactions. This is in line with more general vehicle-VRU literature (Dey and Terken, 2017; Lee et al., 2021), further suggesting a velocity threshold at approximately 25–35 km/h for the relevance of more explicit social cues such as driver gestures or eye contact (Dietrich et al., 2019). Above this threshold, road users will instead try to find appropriate gaps to either cross or merge with the traffic based on implicit cues. Unfortunately, this review did not reveal the more precise role or importance of truck driver-centric cues for facilitating HGV-VRU encounters and interactions.

### 4.2 Interactions Involving Highly Automated HGVs

The second research question has to do with implications of the review for the development of HGVs controlled by highly ADS. From the reviewed studies, we have examples of more fixed communication cues (e.g., more precise type of HGV and VRU characteristics) as well as interactive behaviors that will change rapidly (e.g., road user trajectories, body language). Table 6 contains examples of such cues and behaviors, forming an overview of how information can be derived from a range of channels/mechanisms connected to the road users. More specifically, the table maps possible cues between established categories of communication and the source/origin of information (e.g., vehicle-centric vs. driver-centric). While most of the examples are extracted from the reviewed studies (Section 3), some additions have been made by the authors to provide a more complete view of the possible range of cues these road users might produce and encounter in traffic. From the perspective of the road user, these cues can be classified as either spontaneous (i.e., provided on a nonvoluntary basis), symbolic (i.e., provided deliberately to communicate), or pseudo-spontaneous (seemingly spontaneous cues but with a concealed intentionality) (Buck and VanLear, 2002). This highlights how difficult it might be to differentiate between implicit and explicit communication, and that road users may adopt pseudo-spontaneous strategies to get ahead in traffic (e.g., VRU actively deciding not to look at oncoming traffic put the burden of responsibility on the driver). When considering highly ADS applied to HGVs, Table 6 indicates that these systems will need to handle a wide range of interactive behaviors. Communication based on trajectories and kinematic behavior will form the basis for interaction, but developers of these systems could also find it necessary to compensate for the loss of more social driver-centric cues such as eye-gaze and body language.

**TABLE 6:** Communication channels/mechanisms in HGV-VRU interactions, including examples of extracted road user behaviors/communication cues (regular font) as well as complementary examples added by the authors (italic font).

	HGV		VRUs	
Communication channel/mechanism	Vehicle-centric cues	Driver-centric cues	Pedestrian-centric cues	Cyclist-centric cues
Gestures and movement (kinetics)	HGV adapting trajectory	*Driver hand gesture*	Pedestrian stepping onto the roadway	Cyclist waving
Space (proxemics)	HGV position relative to VRU at an intersection	Driver present inside/outside the truck cabin at loading zone	Pedestrian proximity to other VRUs in the vicinity	Cyclists riding in group
Time (chronemics)	HGV timing acceleration to pass VRU	Driver sequence/order of gaze behavior	Pedestrian initiation of crossing (timing a gap)	Cyclist quickly leaving the near-truck zone
Voice (paralanguage)	*HGV horn sound*	*Driver vocal reaction*	*Pedestrian vocal reaction*	*Cyclist vocal reaction*
Face and eyes (e.g., oculesics)	—	Driver eye-gaze	*Pedestrian facial expression*	Cyclist eye-gaze
Touch (haptics)	HGV producing aerodynamic force on cyclist	—	—	—
Appearance	HGV as a larger vehicle	Professional driver	Young/old pedestrian	Casual cyclist vs. racer
Human-machine interface (HMI)	HGV displaying contents using external HMI (turning indication or other state/intent etc.)	—	—	—
Environment (e.g., olfactics)	*Engine/tire smell*	—	—	—

While these are direct conclusions based on existing HGV-VRU studies, we also expect highly ADS to contribute to shaping future interactions. For example, the addition of this new type of road user could introduce ambiguity regarding who controls the vehicle. They could also lead to reduced perceived risk for interacting VRUs and enhance observability and predictability by providing information made possible by a more consistent and proactive behavior of an ADS compared to a human driver. So, while this review attempts to provide an overview of the current understanding of HGV-VRU interactive behavior, a future frame of reference (and common ground for communication) could be adjusted as VRUs and vehicles controlled by highly automated driving systems get increased experience of interacting with each other.

### 4.3 Limitations and Future Research

Road traffic interaction is challenging to review since it can be connected to multiple perspectives related to the process and outcomes of events in traffic (Section 1.1). For this review, we strictly included peer-reviewed empirical HGV-VRU interaction studies based on primary data sources, and the process was limited to three databases and a range of exclusion criteria (Section 2.2). While it was useful to leverage theoretical concepts and recent definitions of road traffic interaction, data extraction of “interactive behavior phenomena” were limited to a qualitative analysis of the existing studies. During this process, it was often difficult to fully evaluate the presence, motivation, and effect of reported behaviors since many studies had different research motives or units of analysis. Future research could add to our findings by more directly addressing the communicative components of HGV-VRU behaviors. The reviewed studies were linked to three out of the five prototypical interaction scenarios proposed by Markkula et al. (2020) (Section 1.1.2). This is explained by the fact that HGVs and VRUs often have dedicated infrastructure and are coordinated by traffic control devices, resulting in limited points of interaction. Broadening of the scope of the review to include other types of VRUs (e.g., powered two-wheelers) and vehicles could lead to a better understanding of what interaction practices are unique to different constellations of road users. However, to what extent we can generalize between different “types” of road users is not always clear, and discussions will continue of how such categories are best constructed (Holländer et al., 2021).

This study supports the view that traffic encounters and interactions predominantly are reliant on implicit communication, motivating that AV developers need to pay close attention to how even subtle changes in movement and trajectories can affect interactions. Our synthesis suggests that the more precise appearance and design features of a highly automated HGV could be important for how it is perceived by VRUs. Furthermore, while we only found a few examples of explicit driver-centric communication, more general research (e.g., Dietrich et al., 2019) show that such behaviors are primarily to facilitate social low-speed space-sharing scenarios. Future research should investigate the added value for automated HGVs to 1) explicitly signal movement, perception, and appreciation, or 2) request movement and perception from others during such scenarios. The way to support these various effects could be through existing communication mechanisms or by adding new channels such as eHMI based on visual, auditory, or haptic modalities (see eHMI overview in Dey et al., 2020). These modalities can communicate information such as ADS mode, intentions, perception, instructions, commands, advice, and predictions (Habibovic et al., 2018; Schieben et al., 2019; Colley and Rukzio, 2020; Faas et al., 2020).

However, research and development of the interactive capabilities of AVs is complex. The reviewed studies leveraged various controlled and naturalistic approaches to collect both qualitative and quantitative data, and it is evident that the study of road traffic interaction is (and should continue to belong to) a pluralistic research discipline. Within the HGV-VRU delimitation we found only one study addressing highly automated HGV encounters, resulting in the findings being predominantly based on human drivers interacting with (human) VRUs. This indicates that there is need for additional perspectives from fields such as human-computer interaction (HCI). Though HCI research and concepts have been widely used for the in-vehicle experience, AV technology has extended the scope to include traffic interactions. Unfortunately, there are no well-established practices when it comes to investigating or designing for interactions with “AI-infused technology” (Amershi et al., 2019), which at least vehicles controlled by higher levels of driving automation could be based on. Initially, AV-VRU interaction research have largely had to rely on insights from (controlled) experiments using Wizard-of-Oz and VR/simulator approaches, or experiences of early deployments of highly rule-based systems (e.g., AV shuttle buses). However, depending on the architecture of highly automated driving system (and the possible limits in transparency/explainability), any deeper understanding on AV-VRU encounters/interactions could require more naturalistic studies. Analogous to how we study human (or animal) behavior, we might need to study emergent machine behavior (Rahwan et al., 2019) in naturalistic traffic studies. Still, any new perspectives should go together with a deep understanding of existing traffic practices as well as the development of rigorous ways of gathering insights on the subtleties of road traffic interactions (e.g., Markkula et al., 2020; Madigan et al., 2021). In addition, more discussion is needed about what constitutes appropriate road traffic interactions in mixed traffic environments and how those encounters and interactions should be evaluated.

## 5 Conclusion

This systematic review helps to generate an understanding of how drivers of heavy goods vehicles (HGVs) and vulnerable road users (VRUs) interact and communicate with each other, and what it might mean for interactions and communication between highly automated HGVs and VRUs. Overall, it is concluded that discernable interactive behaviors and communication cues from existing HGV-VRU behavioral studies can be categorized in line with concepts from communication theory and the field of road traffic interaction. However, further methodological efforts should be made to support a continued cross-disciplinary understanding of road traffic interaction. Based on our research questions, we conclude the following:

*What interactive road user behaviors and communication cues can be identified in empirical HGV-VRU studies?*

• Like encounters and interactions between other types of road users, HGV-VRU interactions are influenced by interrelated vehicle, individual, and contextual factors.
• Focusing on the road users, we found the following examples of interactive behaviors and communication cues: a) vehicle-centric (e.g., HGV as a larger vehicle, adapting trajectory, positioning relative to the VRU, timing acceleration to pass the VRU, producing aerodynamic force on VRU, displaying information *via* HMI), b) driver-centric (e.g., professional driver, being present inside/outside the cabin, eye-gaze behavior), and c) VRU-centric (e.g., professional vs. casual cyclist, adapting trajectory, positioning relative to the HGV, proximity to other VRUs in the vicinity, timing a gap, gaze behavior, waving to driver).
• Most of the cues that we identified are linked to implicit communication. While it indicates that this is the primary mechanisms for interactions, it could also suggest that the role/importance of communication from HGV driver- and VRU-centric cues (e.g., eye-gaze, facial expressions and vocal reactions) requires additional research. Based on more general literature, such research should focus on low-speed scenarios where this type of communication is more common.
• Another important insight is that HGV drivers commonly adjust their behavior (i.e., gazing, positioning) in areas with VRUs and yield to VRUs even if they have priority. Similarly, VRUs may experience HGVs as threatening, underestimate their speed and adjust (or fail to adjust) their behavior due by blind-spots around HGVs.
• Compared to corresponding scenarios with light vehicles, HGV-VRU interaction patterns are generally formed by the HGV’s size, shape and weight. For example, this can cause VRUs to feel less safe, drivers to seek to avoid unnecessary decelerations and accelerations, or lead to strategic behaviors due to larger blind-spots.
*What are potential implications for future interactions between highly automated HGVs and VRUs?*
• Our conclusions from the first research question indicate that highly automated HGVs might need to handle a wide range of interactive behaviors and communication cues. Road user trajectories and kinematic behavior are likely to form the basis for communication also for highly automated HGV-VRU interaction. However, it might also be beneficial to use additional eHMI to compensate for the loss of more social driver-centric cues or to signal other types of information.
• In particular, developers of highly automated HGVs can try to design their vehicles to appear less threatening. Added eHMI can be used to differentiate them from manually driven HGVs with bigger blind-spots, or to more explicitly signal their perception of VRUs. Also, eHMI could be used to make it easier for VRUs to estimate the speed and distance of the HGV, or to indicate their yielding intentions. The latter might be especially important since it can also reduce the need for abrupt decelerations and accelerations. While this is largely in line with the current knowledge on eHMI for highly automated passenger vehicles, this study provides indications that the value for HGV-VRU interactions could be more pronounced.
• Lastly, these conclusions are based on the existing HGV-VRU studies. We should, however, expect interaction practices to be updated as VRUs and highly automated HGVs get increased experience of interacting with each other.

## Data Availability

The original contributions presented in the study are included in the article/Supplementary Material, further inquiries can be directed to the corresponding author.
